# The Molecular and Cellular Basis of Physiological Changes in Pregnancy and Its Implications in Neurologic and Ophthalmic Pathologies

**DOI:** 10.3390/ijms26115220

**Published:** 2025-05-29

**Authors:** Yi-Ting Chiang, Jie-Hong Chen, Kuo-Hu Chen

**Affiliations:** 1 Department of Medical Education, Taipei Tzu-Chi Hospital, The Buddhist Tzu-Chi Medical Foundation, Taipei 231, Taiwan; 107311109@gms.tcu.edu.tw; 2 Department of Medicine, MacKay Medical College, New Taipei City 25245, Taiwan; albertjhc@gmail.com; 3 Department of Obstetrics and Gynecology, Taipei Tzu-Chi Hospital, The Buddhist Tzu-Chi Medical Foundation, Taipei 231, Taiwan; 4 School of Medicine, Tzu-Chi University, Hualien 970, Taiwan

**Keywords:** physiological changes in pregnancy, neurologic pathology, ophthalmic pathology

## Abstract

Pregnancy orchestrates profound neurological, hormonal, and anatomical transformations in the maternal brain, preparing it for caregiving and infant bonding. Neuroimaging reveals structural changes such as gray matter reductions and white matter reorganization during pregnancy, followed by partial recovery postpartum. These adaptations are modulated by fluctuating levels of estradiol, progesterone, prolactin, and oxytocin, which coordinate neuroplasticity and behavioral readiness. At the molecular and cellular levels, pregnancy hormones drive synaptic remodeling, neurogenesis, and glial activity. Together, these changes support maternal motivation, attachment, and responsiveness, highlighting the maternal brain’s dynamic plasticity across gestation and the postpartum period. Also, pregnancy induces profound physiological changes, particularly in vascular, hormonal, and neurologic systems, to support maternal and fetal health. While these adaptations are essential, they can predispose pregnant individuals to various neurologic and ophthalmic pathologies. This review explores how pregnancy-related changes—including hypercoagulability, pituitary enlargement, hormonal fluctuations, and immunological modulation—contribute to conditions such as stroke, idiopathic intracranial hypertension, preeclampsia-associated visual disturbances, and demyelinating disorders like neuromyelitis optica spectrum disorder and multiple sclerosis. Additionally, ocular manifestations of systemic diseases like diabetic retinopathy and thyroid orbitopathy are discussed. Understanding these complex interactions is critical for prompt recognition, accurate diagnosis, and appropriate management of vision-threatening and neurologically significant complications during pregnancy. Nevertheless, many aspects of physiological and pathological changes during and after pregnancy remain unknown and warrant further investigation.

## 1. Introduction

Pregnancy is a period of remarkable transformation not only physiologically but also neurologically. While many maternal adaptations focus on the cardiovascular, metabolic, and reproductive systems, the maternal brain undergoes equally significant remodeling to support the demands of motherhood. These neurological changes are essential for the onset of caregiving behaviors, bonding with the infant, and adapting to the emotional and physical responsibilities of parenthood [1,2,3].

Recent neuroimaging studies have shed light on the dynamic anatomical changes that occur in the brain during and after pregnancy. Most notably, reductions in gray matter (GM) volume have been consistently observed across several cortical regions, particularly those involved in social cognition, emotion regulation, and maternal motivation [3]. At the same time, pregnancy enhances white matter (WM) microstructure in key neural tracts, potentially increasing neural connectivity and communication across regions that facilitate maternal behavior [4,5,6]. Postpartum, some of these changes begin to reverse or normalize, with volumetric increases in regions such as the hypothalamus, amygdala, and striatum—areas implicated in maternal–infant attachment and affective responsiveness [7,8,9,10].

These structural changes are tightly coupled with the hormonal landscape of pregnancy and postpartum. Steroid and peptide hormones such as estradiol, progesterone, oxytocin, and prolactin play crucial roles in modulating brain plasticity. Their effects extend to the molecular and cellular levels, inducing synaptic remodeling, dendritic growth, neurogenesis, and changes in neurotransmitter systems [3,8]. Animal models further demonstrate that pregnancy hormones can prime the maternal brain for parenting even in the absence of actual pregnancy, underlining the potent neurobiological impact of endocrine shifts. Understanding the molecular and cellular basis of these changes reveals how the maternal brain adapts to meet the complex demands of motherhood. These insights emphasize the intricate interplay between neuroanatomy, hormonal regulation, and maternal behavior that defines the maternal experience during pregnancy and beyond.

Pregnancy is a unique physiological state marked by dynamic and systemic changes across multiple organ systems. To accommodate the growing fetus and prepare for childbirth, the maternal body undergoes significant cardiovascular, hormonal, immunological, and neurological transformations. These adaptations, while necessary, can also unmask, exacerbate, or complicate pre-existing medical conditions, particularly those affecting the nervous system and eyes. As neurologic and ophthalmic structures are sensitive to systemic fluctuations, pregnancy can both initiate de novo pathologies and modulate the course of existing diseases [1,2,3].

Among the most prominent changes is the hypercoagulable state induced by increased coagulation factors and suppressed anticoagulant activity, which enhances the risk of vascular occlusions such as retinal vein thrombosis and cerebral venous sinus thrombosis. The hormonal surges of pregnancy—particularly in estrogen and progesterone—further influence brain volume, vascular tone, and immune regulation. These shifts are implicated in the development of conditions like idiopathic intracranial hypertension, pituitary hyperplasia, and meningioma progression. Additionally, altered cerebral blood flow and sudden postpartum hormonal shifts contribute to a heightened risk of stroke and other cerebrovascular events. Pregnancy also alters the clinical course of autoimmune and inflammatory neurologic disorders. Multiple sclerosis (MS) often stabilizes during gestation due to immunosuppressive modulation but frequently flares postpartum. In contrast, neuromyelitis optica spectrum disorder (NMOSD) poses a higher risk for relapse during and after pregnancy, potentially leading to severe maternal and fetal outcomes. Similarly, systemic disorders such as diabetic retinopathy and Graves’ disease may worsen or present new ophthalmic symptoms due to vascular and hormonal influences [1,2,3,4,5].

Given the wide spectrum of potential complications, a comprehensive understanding of pregnancy-related physiological changes and their neurologic and ophthalmic implications is essential for clinicians. This review synthesizes the current evidence to elucidate these complex relationships and highlight clinical considerations in managing pregnant individuals with neurologic and ocular concerns.

## 2. Methods: Literature Review and Search Strategy

In the current review, the literature has been searched as to solicit basic and clinical research on the underlying molecular and cellular mechanisms of physiological changes and pathological implications during and after pregnancy. Initially, all of the studies were retrieved from the databases Ovid Medline and PubMed using the search terms “physiological changes in pregnancy”, “neurologic pathology”, and “ophthalmic pathology” for the topic of the research, followed by the process of database searching, screening, and selection of the references. In the next stage, only full-text articles were considered for inclusion for further analysis; duplicated articles were also excluded. Furthermore, two experts in the field, respectively, inspected the contents of selected articles and focused on research details, including materials, methods, and results and/or outcomes, to identify eligible studies for subsequent inclusion. Articles with poor research methods, study designs, or mismatched results/outcomes were excluded in this stage. Any discrepancies between these two experts were discussed by mutual communication to reach a consensus. All eligible articles were included in the review using the aforementioned search terms and strategies. Finally, a total of 82 articles were selected for this review.

## 3. The Molecular and Cellular Basis of Physiological Changes in Pregnancy

### 3.1. Antepartum Neuroanatomical Changes in Mothers

Pregnancy-induced neurological changes are essential for preparing the maternal brain to support caregiving behaviors, including nursing, bonding, and responding to infant cues. Neuroimaging studies over the past decade have demonstrated that pregnancy induces significant neuroanatomical changes, leading to visible structural modifications in the maternal brain. MRI-based research has consistently reported volumetric reductions in gray matter (GM), with an average decrease of 3% in total cortical grey matter volume [1]. These reductions are most pronounced in regions strongly associated with theory-of-mind processing and the default mode network, including the prefrontal and temporal lobes, precuneus, insula, anterior cingulate cortex, and medial temporal areas such as the hippocampus and parahippocampal gyrus [2,3].

Longitudinal analyses of first-time mothers have shown pronounced reductions in cortical thickness, surface area, local gyrification, sulcal depth, and sulcal length, along with an increase in sulcal width. In contrast to the reduction in gray matter volume during pregnancy, white matter (WM) microstructural integrity increases across the brain. This enhancement is observed in several major tracts, including the corpus callosum, arcuate fasciculus, inferior fronto-occipital and inferior longitudinal fasciculi, cingulum bundle, middle and superior longitudinal fasciculi, as well as the corticostriatal, corticospinal, and corticopontine tracts [4]. Particularly, inferior longitudinal fasciculus and inferior fronto-occipital fasciculus is associated with facilitating communication between emotional and visual processing hubs [4].

Additionally, white matter volume increases have been observed in frontal, temporal, and superior parietal regions as well as subcortical structures like the thalamus, caudate, and cerebellum, with these alterations being positively correlated with maternal empathic abilities. These pre-to-post pregnancy cortical changes have been associated with increased quality of mother-to-infant attachment and reduced likelihood of maternal hostility toward the infant [2]. Furthermore, a reduction in nucleus accumbens volume during pregnancy has been positively correlated with heightened neural responses to images of their infant after delivery [5].

### 3.2. Postpartum Neuroanatomical Changes in Mothers

While the initial postpartum period is characterized by widespread reductions in GM volume, some of these changes show partial recovery over time [6]. Mothers exhibit significantly lower GM volume 1–2 months after childbirth compared to nulliparous women; however, this difference progressively declines over the following 3–4 months as GM volume gradually restores in multiple brain regions [7,8,9]. Pregnancy-related reductions in brain volume undergo a process of renormalization during the first months postpartum rather than reflecting additional structural gains [7]. Key regions involved in maternal motivation and behavior, including the hypothalamus, amygdala, substantia nigra, and globus pallidus, exhibit increased GM volume with time during early postpartum period [10]. These structural changes appear to be influenced by maternal–infant bonding, as a mother’s positive perception of her baby within the first month predicts subsequent GM volume increases in these areas [10]. Maternal–newborn interactions play a role in the remodeling of maternal neuroanatomical structure. Many of the brain regions that show GM volume reductions during pregnancy overlap with those that demonstrate volume increases in the postpartum period. These include areas involving the precuneus, superior temporal gyrus, inferior frontal cortex, medial prefrontal cortex, anterior cingulate cortex, medial temporal structures (hippocampus, parahippocampal gyrus, and insula), as well as visual areas (middle occipital gyrus, posterior cerebellum, and both ventral and dorsal striatal regions, including the nucleus accumbens and caudate) [3]. Furthermore, significant increases in WM microstructural integrity during pregnancy are observed to return to baseline levels in the early postpartum period [4].

Thus, early postpartum neuroplasticity not only facilitates recovery from pregnancy-related GM reductions but is also shaped by maternal affective and motivational processes. Table 1 shows a summary of structural and functional changes in brain regions during pregnancy and postpartum.

### 3.3. The Role of Pregnancy Hormones in Anatomy

The shift from pregnancy-related gray matter reductions to postpartum increases corresponds with the dramatic hormonal changes occurring before and after delivery [3]. Steroid hormones, including estradiol, progesterone, and cortisol, play a crucial role in regulating neurodevelopmental processes during pregnancy, orchestrating structural and functional brain changes in the maternal brain. Pregnancy-related hormones such as steroid hormones (estradiol, progesterone, and corticosterone) and peptide hormones (oxytocin and prolactin) collectively facilitate and regulate maternal behavior [3]. Mainly secreted from the ovaries, estradiol has the strongest potency among all estrogens (estrone (E1); estradiol (E2); estriol (E3)) and dominates the female reproductive system in non-pregnant women. However, the growing placenta during pregnancy can replace the ovaries, functioning as the main endocrinal organ to produce and synthesize estriol (E3) and progesterones. Greater reductions in total gray matter volume during pregnancy are linked to higher circulating estradiol levels in the third trimester (gestation 28–40 weeks) [8]. During and after pregnancy, the peptide hormones oxytocin and prolactin play vital roles in shaping maternal physiology and behavior. Oxytocin, produced in the hypothalamus and released by the posterior pituitary gland, is well known for its role in uterine contractions during labor and milk ejection during breastfeeding. Beyond these functions, oxytocin exerts significant effects on the maternal brain, promoting bonding and social behavior by enhancing activity in brain regions associated with emotion and reward. It supports maternal–infant attachment and may reduce anxiety, fostering a calm and nurturing state. Prolactin, secreted by the anterior pituitary gland, is essential for the initiation and maintenance of lactation, stimulating milk production in the mammary glands. It also influences maternal motivation and caregiving behavior by acting on key brain regions such as the hypothalamus and amygdala. Elevated prolactin levels during postpartum have been associated with increased parental responsiveness and protective behavior. Together, oxytocin and prolactin coordinate complex changes in the neurological, endocrine, and reproductive systems to support maternal functions, enhance caregiving, and reinforce the mother–infant bond during and after pregnancy [3,8].

### 3.4. Hormonal-Mediated Neuroplasticity

**Figure 1:**
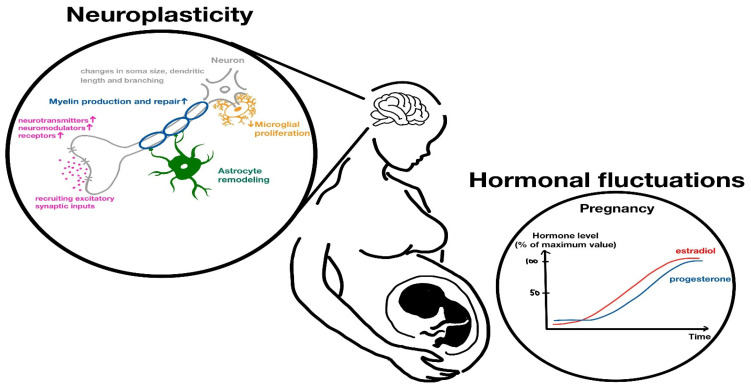
A schematic diagram of a pregnant woman, highlighting the key neurological changes observed during pregnancy, including the affected brain regions and the role of hormonal factors.

**Figure 2:**
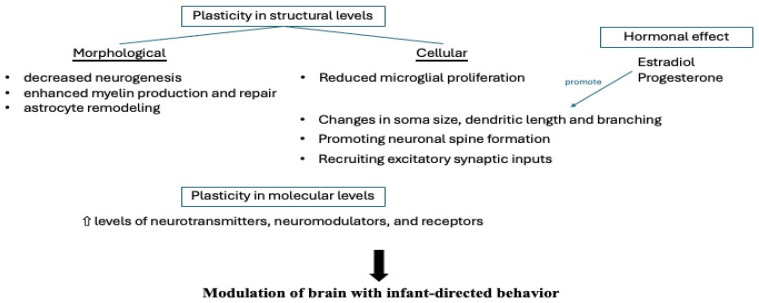
Neuroplasticity at structural, cellular, molecular, and hormonal levels modulating maternal brain for infant-directed behavior during pregnancy.

Hormonal fluctuations associated with motherhood play a crucial role in pregnancy-induced neuroplasticity. During gestation, the up-regulation of steroid hormone synthesis contributes to neurogenesis, dendritic spine formation, microglial proliferation, myelination, and astrocyte remodeling [4]. These neurobiological changes are particularly evident in brain circuits involved in maternal behaviors [4]. Studies in rodent models indicate that pregnancy hormones, such as estradiol, progesterone, corticosterone, prolactin, and oxytocin, largely drive these neuroplastic changes, as parental behavior can be induced in virgin rats by administrating similar hormones and simulating pregnancy-related hormonal changes—particularly the significant elevation of estradiol and progesterone—suggesting that such hormonal modulation primes the brain for infant-orientated behavior [11]. Figure 1 is a schematic diagram of a pregnant woman that highlights the key neurological changes observed during pregnancy, including the affected brain regions and the role of various hormonal factors. Figure 2 depicts the modification (neuroplasticity) at structural, cellular, molecular, and hormonal levels modulating maternal brain for infant-directed behavior during pregnancy.

In rodents, steroid hormones enhance maternal responsiveness to offspring by modulating galanin-expressing neurons in the medial preoptic area (mPOA) of the hypothalamus. Galanin-expressing neurons in the mPOA are observed to be involved in regulating the motor, motivational, hormonal, and social components of parenting [12].

**Figure 3:**
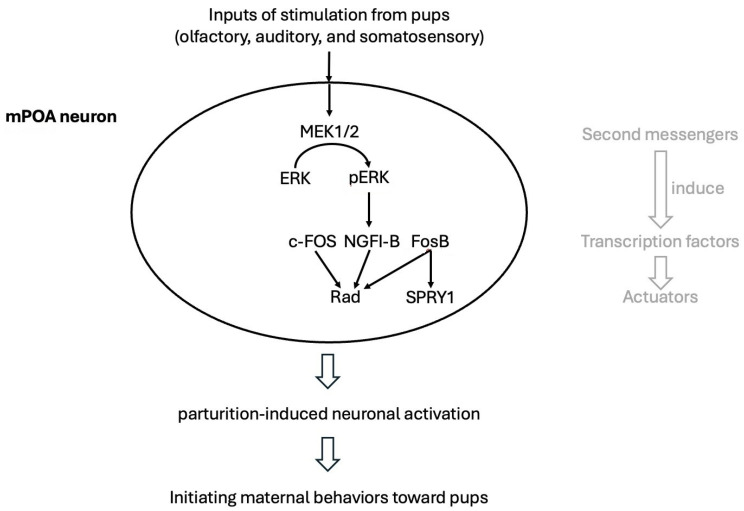
The physiological adjustment mechanisms of mPOA neurons involved in the development of hormonal mediated neuroplasticity, illustrated with the cascade of protein kinases where MEK phosphorylates and activates ERK. mPOA: medial preoptic area; MAPK: mitogen-activated protein kinases; ERK: extracellular signal-regulated kinase; MEK: MAPK/ERK kinase; c-FOS: cellular Fos proto-oncogene, AP-1 transcription factor subunit; NGFI-B: nerve growth factor-inducible gene B; SPRY1: Protein sprouty homolog 1.

**Figure 4:**
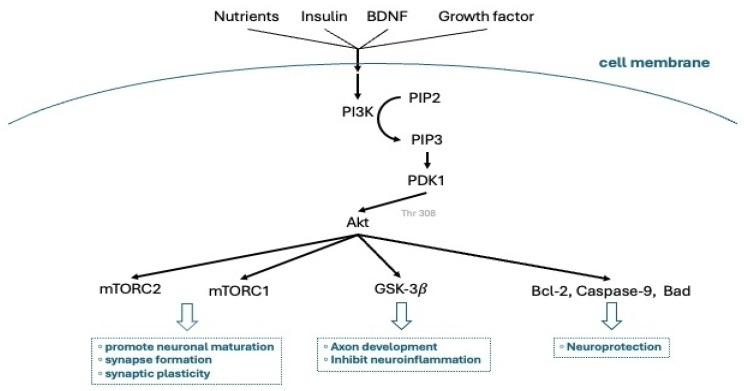
The physiological mechanisms of neuronal maturation, axonal development, and neuroprotection in response to effects of external factors (nutrients, insulin, and growth factors), mediated by PI3K and Akt molecular pathways. BDNF: brain-derived neurotrophic factor; PI3K: phosphoinositide 3-kinases; PDK1: pyruvate dehydrogenase kinase 1; Akt: protein kinase B; PIP2: phosphatidylinositol 4,5-bisphosphate; PIP3: phosphatidylinositol (3,4,5)-trisphosphate; GSK-3β: glycogen synthase kinase 3β; mTORC: mammalian target of rapamycin complex; Bcl-2: B-cell leukemia/lymphoma 2.

Hormone-mediated neuroplasticity in the maternal rodent brain occurs at multiple levels, encompassing molecular, cellular, and morphological changes. These hormonally driven adaptations are mediated by cytoplasmic kinase cascades and genomic signaling pathways, leading to two forms of neuroplasticity: molecular and structural. Estradiol and prolactin, acting through specific receptors in the mPOA, activate these intracellular signaling pathways, ultimately generating functionally active, pup-responsive mPOA neurons [13,14,15]. Cytoplasmic kinase cascades and genomic signaling pathways act as key mediators of neuroplasticity during pregnancy by translating hormonal signals into functional changes within neurons. When hormones like estradiol and prolactin bind to their respective receptors in the medial preoptic area (mPOA), they initiate kinase cascades such as the MAPK/ERK (Figure 3) and PI3K/Akt (Figure 4) pathways. These signaling pathways rapidly alter intracellular activity by phosphorylating target proteins involved in cell growth, survival, and synaptic plasticity. Concurrently, these cascades often converge on transcription factors—such as CREB and STAT5—which then translocate to the nucleus to regulate gene expression, resulting in long-term genomic effects. This dual action gives rise to two interconnected forms of neuroplasticity. Molecular neuroplasticity involves changes in gene transcription, protein synthesis, and receptor expression that enhance neuronal excitability and responsiveness to pup stimuli. Structural neuroplasticity refers to morphological changes such as dendritic branching, synapse formation, and remodeling of glial cells. Together, these processes increase the sensitivity and efficiency of maternal brain circuits, particularly within the mPOA, to support caregiving behaviors and promote maternal–infant bonding throughout gestation and postpartum [13,14,15].

Structural plasticity includes alterations in neuronal and glial morphology as well as fluctuations in cell numbers, manifesting as decreased neurogenesis, reduced microglial proliferation, and enhanced myelin production and repair. Compared with nulliparous rats, pregnant rats exhibit significant structural plasticity, including changes in the soma size, dendritic length, branching, and spine density of mPOA neurons [16]. At the molecular level, neuroplasticity involves increased levels of neurotransmitters, neuromodulators, and receptors, which modulate neural electrical activity [3]. Additionally, throughout pregnancy, estradiol and progesterone facilitate the gradual formation of perineuronal nets in the mPOA—extracellular matrix structures that stabilize synaptic connections during phases of neuroplasticity [17]. These pregnancy-related hormonal influences may promote dendritic growth and reinforce neural circuits within the maternal brain, particularly in late gestation. Furthermore, estradiol and progesterone play a crucial role in regulating galanin-expressing neurons within the mPOA, a process essential for pregnancy-induced parental behavior. While estradiol paradoxically silences these neurons, it simultaneously increases their excitability. On the other hand, progesterone permanently remodels the circuit by promoting dendritic spine formation and recruiting excitatory synaptic inputs. This mPOA-specific neural restructuring reduces population activity in vivo, leading to more selective and robust responses to pup stimuli. Consequently, pregnancy hormones reshape maternal neural circuits, priming the brain for caregiving behaviors postpartum [11].

### 3.5. Neurogenesis

Rats in the peripartum period are marked by changes in neurogenesis within the dentate gyrus of the hippocampus and the subventricular zone of the lateral ventricles [18]. During pregnancy, surges in prolactin promote neurogenesis in the subventricular zone, with newly generated neurons migrating to the olfactory bulb, potentially enhancing odor recognition and facilitating maternal behavior [19].

Nevertheless, studies indicate that hippocampal cell proliferation declines in maternal rats compared to nulliparous rats, particularly during the transition to motherhood. The reduction gradually progresses from the mid-postpartum period onwards, accompanied by decreased survival of proliferating hippocampal cells [20,21,22,23,24,25,26]. The decline in hippocampal neurogenesis during the postpartum period has been linked to the sharp drop in estradiol levels and persistently elevated corticosterone levels associated with lactation [20,22,23,24]. These findings suggest that peripartum steroid hormone fluctuations may suppress hippocampal neurogenesis from mid-pregnancy to the late postpartum period in the dam.

## 4. The Implications of Pregnant Physiological Changes in Neurologic and Ophthalmic Pathologies

From the beginning of pregnancy, vascular and neurologic changes can be found. During pregnancy, a decrease in brain volume and a slight enlargement of the ventricles are observed on brain MRI, likely influenced by pregnant hormonal changes and alkalosis. These alterations appear to be temporary, as brain volume gradually returns to normal after delivery. Biochemical and hormonal shifts during and after pregnancy are believed to support maternal adaptation for motherhood. However, these pregnant physiological changes may have pathologic implications for a vascular change (hypercoagulable status) or event (cerebral stroke) and other neurological or ophthalmic complications.

### 4.1. Vascular Change—Hypercoagulable Status

Starting in early pregnancy, cardiac output and blood volume increase by 30–50%, accompanied by a hypercoagulable state due to decreased fibrinolytic activity and increased levels of plasminogen, fibrinogen, and clotting factors I, V, VII, IX, and X, while the activities of antithrombin III, protein S, and protein C are inhibited [27,28]. This physiological adaptation predisposes pregnant individuals to vascular occlusive diseases, including retinal artery and vein occlusions, disseminated intravascular coagulation (DIC), thrombotic thrombocytopenic purpura (TTP), antiphospholipid antibody syndrome (APS), amniotic fluid embolism, and cerebral venous sinus thrombosis [29].

Among these vascular occlusive diseases, DIC, often triggered by pregnancy complications (e.g., preeclampsia and amniotic fluid embolism), leads to choriocapillaris thrombosis in the retinal pigment epithelium, resulting in serous retinal detachment. HELLP syndrome, frequently coexisting with DIC, is also associated with serous retinal detachment, vitreous hemorrhage, and central retinal vein occlusion. TTP, though rare, presents ocular changes in 10% of cases, including serous retinal detachment, retinal hemorrhage, exudates, and arteriole narrowing. APS, a thrombophilic disorder, increases the risk of vascular thrombosis, affecting not only the anterior and posterior segments but also the optic nerve, potentially leading to significant visual complications [29,30].

### 4.2. Vascular Event—Cerebral Stroke

Pregnancy increases the risk of cerebrovascular disease by 3–13 times in healthy young women, with the stroke risk estimated at 0.7 during pregnancy but rising significantly to 8.7 in the postpartum period [31]. During pregnancy, rising progesterone levels cause blood vessel dilation and reduced venous return. Postpartum, the sudden drop in progesterone may lead to cerebral vasoconstriction, increasing the risk of cerebral ischemia and, in severe cases, ischemic stroke, contributing to the high incidence of postpartum stroke [32,33]. Several factors contribute to the risk of stroke in pregnancy, including hypertension, fluid and electrolyte imbalances, acid–base disturbances, and cesarean delivery [34]. The pathogenesis of ischemic stroke in pregnancy is mainly driven by hypercoagulability, along with cerebral arterial spasm and endothelial damage caused by impaired organ blood supply, leading to vascular obstruction and ischemia [35]. By the late second trimester (gestation 14–27 weeks), cardiac output, blood volume, and arterial pressure increase, further raising the risk of intracranial hemorrhage, particularly during labor and delivery. Additionally, pregnancy heightens the likelihood of hemorrhage from pre-existing arteriovenous malformations (AVMs), cavernous hemangiomas, and intracranial aneurysms [35,36]. Due to hyperdynamic circulation state and coagulation changes during pregnancy, ischemic strokes affecting the visual or ocular motor pathways can result in neuro-ophthalmic complications.

### 4.3. Pituitary Gland and Pituitary Adenoma

During pregnancy, the pituitary gland undergoes physiologic hyperplasia, primarily due to the proliferation of prolactin-producing lactotroph cells, which are stimulated by elevated estrogen and progesterone levels [37]. This leads to a gradual increase in serum prolactin, which parallels the enlargement of the pituitary gland. The lactotroph hyperplasia begins early in pregnancy and typically resolves spontaneously within 2 weeks to 6 months postpartum [31,38].

Although physiologic pituitary enlargement generally remains confined within the sella turcica and rarely causes symptoms, cases of optic chiasm compression have been reported, leading to diplopia, headaches, blurred vision, and other neuro-ophthalmic symptoms [39,40,41]. In contrast, pre-existing pituitary adenomas, particularly prolactinomas, may enlarge significantly during pregnancy, as their estrogen receptor expression makes them highly responsive to hormonal stimulation. While microadenomas (<1 cm) rarely cause optic chiasm compression, macroadenomas carry a higher risk of vision impairment because of their potential for extrasellar expansion [42].

### 4.4. Meningioma

Meningiomas can exhibit more aggressive behavior during pregnancy. During pregnancy, meningiomas manifest distinct characteristics compared to those found in the general population, particularly in their tendency to develop near the parasellar region, reliance on anterior circulation for blood supply, and increased likelihood of causing visual disturbances upon diagnosis, and histologically, they are more frequently identified as clear cell or chordoid subtypes [43]. Elevated levels of estrogen, progesterone, and prolactin during pregnancy contribute to tumor growth and vascularization, with vascular endothelial growth factor (VEGF) further promoting expansion [43,44]. The most significant tumor enlargement typically occurs during the second and third trimesters (gestation 28–40 weeks), potentially leading to optic nerve compression and visual impairment [34]. While some tumors regress postpartum, they may recur or enlarge in subsequent pregnancies [45].

### 4.5. Preeclampsia and Eclampsia

Visual disturbances are common in pregnancies that are complicated with preeclampsia and eclampsia, with manifestations including serous retinal detachment, color vision deficits, cranial nerve palsies, and, in severe cases, blindness. These symptoms primarily result from hypertensive retinopathy, characterized by retinal arteriolar narrowing, hemorrhages, and cotton wool spots as well as focal choroidal ischemia leading to Elschnig spots and serous retinal detachment [31,35,46]. In severe cases, retinal vasospasm and nonarteritic ischemic optic neuropathy (NAION) may occur, while optic disc edema can develop due to increased intracranial pressure. Additionally, cortical visual impairment, including transient or permanent vision loss, may result from posterior reversible encephalopathy syndrome (PRES) or hypertensive stroke [34,47]. The primary treatment for severe preeclampsia and eclampsia is prompt delivery; however, intravenous magnesium sulfate is often administered to prevent seizures, alleviate cerebral vasospasm, and stabilize the blood–brain barrier [48]. Notably, magnesium sulfate may also cause neuro-ophthalmic side effects, such as impaired accommodation, convergence deficits, and ptosis [49,50,51].

### 4.6. Papilledema

Idiopathic intracranial hypertension (IIH) is characterized by increased intracranial pressure without an underlying infection, inflammation, thrombosis, or mass lesion. In pregnant individuals, particularly those who are overweight or obese, IIH can lead to neuro-ophthalmic symptoms, including blurred vision, papilledema, and pulsatile tinnitus. Some patients may also experience diplopia due to sixth cranial nerve palsy. The severity of vision loss varies, ranging from mild transient disturbances to permanent impairment, depending on the degree of papilledema and disease progression. While pregnancy does not increase the overall incidence of IIH, hormonal fluctuations and pregnancy-related weight gain can exacerbate symptoms in individuals with preexisting IIH [34].

### 4.7. Migraine

Estrogen plays a significant role in migraine development, which may explain why migraines are more prevalent in women than in men [52]. Migraine commonly affects women during childbearing years and may first present during pregnancy [28,53]. For some affected females, a worsening of migraine may be the early indication of pregnancy [54]. However, migraine symptoms typically improve during pregnancy, especially in the second and third trimesters (gestation 14–40 weeks), though a small subset of individuals, particularly those with a history of migraine aura, may experience worsening of the symptoms [54].

The hormonal influence on migraines is evident during pregnancy, the postpartum period, perimenopause, and menopause. In an experimental study, exogenous estrogen and progesterone exposure in ovariectomized mice increased cortical spreading depression, which may explain why migraine aura worsens during pregnancy [54]. Migraine aura can emerge as estrogen levels rise in pregnancy but also frequently appears postpartum when estrogen levels drop rapidly, suggesting that fluctuations in estrogen levels may act as a migraine trigger.

### 4.8. Cranial Neuropathy

Cranial neuropathies can develop during pregnancy, with Bell’s palsy being the most common, followed by sixth and fourth cranial nerve involvement [55]. More than two-thirds of affected cases occur in the third trimester (gestation 28–40 weeks) or postpartum period, likely due to increased interstitial fluid leading to nerve compression [31]. Pregnancy-related Bell’s palsy tends to be more severe, with a higher risk of long-term complications, such as complete facial paralysis in pregnant women, compared to nonpregnant women and men [56].

### 4.9. Diabetic Retinopathy

Pregnancy is a risk factor for the progression of diabetic retinopathy (DR), with additional contributors including diabetes duration, metabolic control, baseline retinopathy severity, hypertension, age of onset, visual acuity, and diabetic macular edema [57,58]. Pregnant women with pre-gestational (overt) or gestational diabetes are at risk of developing or worsening nonproliferative and proliferative diabetic retinopathy, with progression often occurring during pregnancy but regressing postpartum [28,58,59,60]. The pathogenesis of DR in pregnancy is driven by vascular, hormonal, and inflammatory changes that accelerate disease progression. Increased cardiac output and impaired ability of autoregulation lead to retinal vessel dilation and altered capillary perfusion, while placental growth hormone and insulin-like growth factor promote angiogenesis. Additionally, up-regulation of pro-inflammatory factors accompanied with down-regulation in anti-inflammatory factors, reduced glycoprotein glycodelin, and elevated endothelin-1 contribute to endothelial dysfunction, worsening nonproliferative diabetic retinopathy or progression to proliferative diabetic retinopathy [57].

### 4.10. Neuromyelitis Optica Spectrum Disorder (NMOSD)

Neuromyelitis optica spectrum disorder (NMOSD) is a rare, antibody-mediated inflammatory demyelinating disorder of the central nervous system, characterized by core clinical features such as optic neuritis, transverse myelitis, brainstem syndrome, and area postrema syndrome [61].

NMOSD is significantly more prevalent in women than in men, with female-to-male ratios ranging from 3:1 to 10:1 and a mean age of onset between 34 and 43 years [62,63]. Consequently, a substantial proportion of patients are women of childbearing age at disease onset [64].

**Figure 5:**
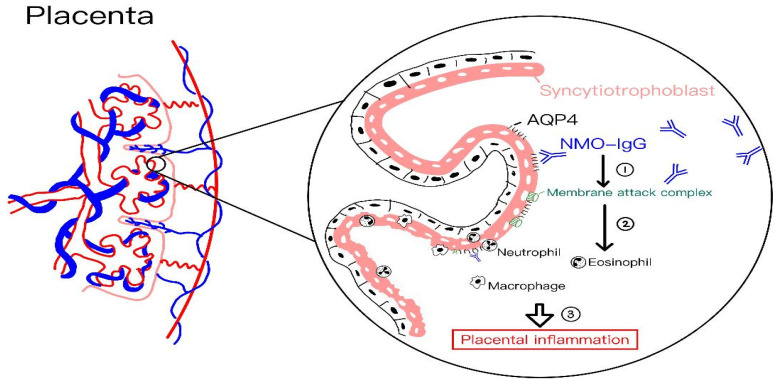
The proposed pathophysiology of placental inflammation in pregnant women with NMOSD. ① The binding of NMO-IgG to AQP4 leads to the activation of complement, resulting in deposition of membrane attack complexes. (②, ③) Leukocytes (including neutrophils, macrophages, and eosinophils) infiltrate and release elastase and proteases, causing placental inflammation and damage.

Aquaporin-4 (AQP4), the main target antigen in NMOSD, is highly expressed in the healthy placenta, suggesting its involvement in maintaining maternal–fetal fluid balance, electrolyte stability, and overall water homeostasis throughout pregnancy [65]. The levels of autoantibody AQP4-IgG reach a peak during mid-gestation and gradually decline as pregnancy progresses, which may contribute to a greater risk of miscarriage during periods of active disease [66]. Simultaneously, the onset of NMOSD after pregnancy is an independent risk factor for miscarriage. Figure 5 illustrates the proposed pathophysiology of placental inflammation in pregnant women with NMOSD.

A proposed mechanism studied in a mouse model suggests that AQP4 antibodies bind to syncytiotrophoblasts of the placental villi, triggering classical complement activation and leading to C5b-9 deposition, AQP4 expression loss, and damage to syncytiotrophoblasts. This is followed by leukocyte infiltration—primarily neutrophils—which release proteolytic enzymes such as elastase, causing further placental injury. In severe cases, extensive inflammation results in necrosis and fetal loss, whereas milder inflammation appears compatible with normal fetal survival and birth [66]. Furthermore, the relapse rate increases not only during pregnancy but also during the postpartum period [67,68,69,70], implicating the long-term effect of AQP4-IgG complex and its activation in the circulation system even if the placenta is removed after delivery. Although AQP4 antibodies can cross the placenta and be detected in neonatal circulation, they typically do not cause clinical symptoms in newborns. However, NMOSD may occur in the maternal central nervous system to manifest neurological and optical damages after inflammation and demyelination of nerve tissues.

### 4.11. Multiple Sclerosis (MS)

Women with multiple sclerosis (MS) generally experience fewer attacks of optic neuritis during pregnancy, as MS activity tends to decrease both clinically and in MRI scans, particularly in the third trimester (gestation 28–40 weeks). This improvement is thought to result from pregnancy-induced immunological modulation, characterized by a relatively immunosuppressed state [71]. Elevated levels of estrogens and other sex hormones shift the T-helper (Th) cell response toward a Th2-dominant profile—associated with anti-inflammatory cytokine production—while suppressing the Th1 pro-inflammatory response [72]. Notably, this immunological balance is reversed postpartum. Eventually, a significant rebound in relapse rate of MS is often observed in the early postpartum period [73,74]. Although both MS and NMOSD tend to show increased disease activity in the postpartum period, this relative remission is less pronounced in patients with NMOSD, as relapse severity and accumulated disability following pregnancy appear to be significantly greater in NMOSD [47,70,75]. Given that MS does not significantly influence overall disease progression or markedly increase complications or affected fetal outcomes, pregnancy is not generally considered high risk in women with MS [72].

### 4.12. Graves’ Disease and Thyroid Orbitopathy

Human chorionic gonadotropin (hCG), a glycoprotein hormone produced by the placenta, shares its alpha subunit with thyroid-stimulating hormone (TSH) but has a distinct beta subunit. Acting as a partial TSH agonist, elevated hCG in early pregnancy can stimulate the thyroid and contribute to transient gestational thyrotoxicosis, which typically occurs during the first trimester (gestation 0–13 weeks). Correspondingly, free thyroxine (FT4) levels transiently increase in the first trimester (gestation 0–13 weeks) because of elevated concentration of circulating hCG and then decline during the second and third trimesters (gestation 14–40 weeks) while generally remaining within the normal range [76]. These physiological changes in maternal thyroid function are closely linked to the increased demand for T4 needed for fetal development, particularly for neuronal development.

Graves’ disease tends to be aggravated in the first trimester (gestation 0–13 weeks), and ameliorated in the second and third trimesters (gestation 14–40 weeks), and commonly relapses postpartum [77]. This pattern of disease progression and regression may be explained by both hormonal and immunological factors. In the first stage of gestation, the initial aggravation of severity of Graves’ disease may result from rising levels of thyroid receptor antibodies (TRAbs) in combination with hCG-driven thyroid stimulation. In contrast, the improvement in disease activity observed after the first trimester (gestation 0–13 weeks) may be attributed to pregnancy-induced immunomodulation [76]. Pregnancy represents a unique immunological state characterized by a functional shift from a T-helper 1 (Th1)-dominated response to a T-helper 2 (Th2) bias, which promotes maternal immune tolerance and protects the fetus from maternal cell-mediated immune reactions [78]. Several pregnancy-associated factors, including leukemia inhibitory factor, progesterone, progesterone-induced blocking factor, and estradiol, are known to support this Th2-dominant state [79].

Graves’ hyperthyroidism is the most frequent underlying cause of thyroid orbitopathy, which often shows clinical improvement during pregnancy but tends to recur after delivery [34]. TRAbs levels tend to decrease throughout pregnancy progress due to the physiological immunosuppression, as TRAbs are detectable in the first trimester (gestation 0–13 weeks), but their levels decrease after 20 weeks of gestation, becoming undetectable toward the term of pregnancy [80]. To sum up, the clinical course of Graves’ disease during pregnancy is variable. While TRAb levels generally decline during gestation and rise postpartum, this pattern is fluctuating and not uniform [81]. Notably, the trajectory of thyroid autoantibodies does not always correlate with orbital manifestations. In particular, antibodies associated with orbital immune responses—such as calsequestrin and collagen XIII—exhibit only slight, nonsignificant reductions during pregnancy and occasional rebound postpartum, without consistent association with the severity of ocular symptoms [82]. Hence, the progression of ocular involvement may not consistently mirror that of thyroid autoimmunity [81]. Table 2 is a summary of the impact of pregnancy on existing ophthalmic disorders.

## 5. Discussion

Pregnancy often brings significant psychological changes influenced by external stressors such as loss of work, reduced privacy, and increasing time demands. The transition to motherhood can heighten emotional sensitivity, and these factors may intensify feelings of anxiety, uncertainty, or loss of control. Financial strain from job loss can increase stress, while the growing physical and emotional needs during pregnancy can limit personal time and privacy, impacting mental well-being. The anticipation of parenting responsibilities may also lead to feelings of overwhelm or self-doubt. These psychological shifts, if not addressed, can contribute to prenatal depression or anxiety, affecting both maternal and fetal health.

Also, external influences such as environmental exposures and maternal lifestyle choices can significantly impact pregnancy outcomes at various stages. Factors like smoking, alcohol consumption, poor nutrition, and exposure to toxins or infectious agents can interfere with fetal development and placental function. In early pregnancy, these influences may lead to miscarriage or congenital anomalies, while in later stages, they can contribute to complications such as preterm labor, low birth weight, or gestational hypertension. Additionally, chronic stress and lack of prenatal care can further compromise maternal and fetal health.

Premature delivery can significantly disrupt the natural progression of a mother’s physical, emotional, and psychological transition into parenthood. The early birth of an infant often means the mother must shift abruptly from pregnancy to intensive caregiving, bypassing important developmental stages such as emotional bonding and preparation for motherhood. This sudden transition can increase the risk of postpartum depression, anxiety, and long-term stress. Additionally, the prolonged hospitalization of the infant and uncertainty about the child’s health can strain maternal–infant attachment. Over time, these challenges may impact the mother’s confidence, coping ability, and overall mental well-being, influencing long-term maternal and family outcomes. Understanding and managing these external factors is crucial for supporting a healthy pregnancy and birth.

Pregnancy initiates a cascade of neuroanatomical and neuroplastic changes in the maternal brain, shaped by hormonal fluctuations and essential for caregiving behaviors. Antepartum gray matter (GM) reductions, particularly in areas involved in social cognition and emotional processing, likely reflect adaptive synaptic pruning, enhancing maternal sensitivity to infant cues. Simultaneously, white matter (WM) integrity increases in key tracts, potentially supporting efficient communication across brain networks [3,4,5,6]. Postpartum, many GM reductions partially reverse, especially in brain regions mediating reward, motivation, and emotional regulation—highlighting the role of maternal–infant interactions in sculpting the maternal brain [7,8,9]. These changes are hormonally driven; estradiol, progesterone, oxytocin, and prolactin orchestrate both molecular and structural neuroplasticity via kinase cascades and genomic signaling pathways [3,8]. Rodent models demonstrate how these hormones remodel the medial preoptic area (mPOA), a central hub for maternal behavior, by enhancing galanin-expressing neuron responsiveness. Neurogenesis in the subventricular zone, stimulated by prolactin, supports olfactory-mediated maternal behaviors, though hippocampal neurogenesis declines due to hormonal shifts in the postpartum period [12,13,14,15]. Collectively, these complex, hormone-mediated changes enable rapid behavioral adaptations, reinforcing maternal responsiveness, bonding, and caregiving. The findings underscore the dynamic interplay between endocrine function, neural structure, and behavior across the peripartum period.

Pregnancy induces widespread physiological changes, particularly hormonal, vascular, and immunological, that significantly influence neurologic and ophthalmic pathologies. The hypercoagulable state [27,28,29] of pregnancy increases susceptibility to vascular occlusive events, such as DIC, APS, and cerebral venous thrombosis, with implications for both vision and brain function [29,30]. Ischemic and hemorrhagic strokes are more prevalent, especially postpartum, due to hemodynamic shifts and endothelial dysfunction [31,32,33]. Similarly, pre-existing neurologic and ophthalmic disorders, including pituitary adenomas, meningiomas, and diabetic retinopathy, may worsen during pregnancy owing to hormonal stimulation and vascular dysregulation [37,38,43,58]. Conditions like IIH, migraine, and cranial neuropathies are also exacerbated by fluid retention and hormonal flux [31,34,54]. Autoimmune disorders, such as NMOSD and MS, show contrasting pregnancy responses: MS typically improves during gestation but rebounds postpartum, whereas NMOSD may remain active throughout and poses risks to maternal and fetal health [61,71]. Endocrine-related conditions, such as Graves’ disease and thyroid orbitopathy, demonstrate fluctuating courses due to the dynamic immunosuppressive state of pregnancy. Notably, ocular complications—ranging from serous retinal detachment to optic neuropathies—are not merely secondary manifestations but often serve as key indicators of underlying systemic pathology. Thus, multidisciplinary care is essential to mitigate maternal and fetal morbidity while safeguarding visual and neurologic integrity during pregnancy.

## 6. Conclusions

Pregnancy and the postpartum period drive profound, hormone-mediated neuroanatomical changes that prepare the maternal brain for caregiving. Structural remodeling—including gray matter reduction, white matter enhancement, and neurogenesis—occurs across key brain regions involved in empathy, motivation, and attachment. These adaptations are guided by fluctuating levels of estradiol, progesterone, oxytocin, and prolactin, which coordinate molecular and cellular plasticity. Maternal–infant interactions further refine brain architecture postpartum, supporting emotional bonding and behavioral responsiveness. Understanding these dynamic changes offers critical insights into maternal brain function, with implications for improving maternal mental health and developing interventions for postpartum neuropsychiatric conditions.

Pregnancy presents a unique physiological landscape that can both exacerbate and modulate neurologic and ophthalmic pathologies. The dynamic interplay of hormonal, vascular, and immunological changes not only alters disease progression but also introduces new risks, including stroke, visual impairment, and autoimmune flare-ups. Awareness of these implications is critical for timely diagnosis, interdisciplinary management, and minimizing adverse maternal–fetal outcomes. Individualized risk assessment, close monitoring, and preconception counseling are essential for patients with pre-existing conditions. Ultimately, a nuanced understanding of pregnancy-related physiological shifts enables clinicians to anticipate complications and tailor care strategies to ensure optimal maternal and neonatal health. Nevertheless, many aspects of physiological and pathological changes during and after pregnancy remain unknown and warrant further investigation.

## Figures and Tables

**Table 1:** Structural and functional changes in brain regions during pregnancy and postpartum.

Brain Region	Observed Changes During Pregnancy	Clinical and Functional Implications	Observed Changes Postpartum
Cortical gray matter	Average volumetric reduction (~3%); Marked decrease in cortical thickness, surface area, local gyrification, sulcal depth, and sulcal length; Increased sulcal width; Network-level changes in: (1) Medial prefrontal cortex (2) Anterior cingulate cortex (3) Lateral prefrontal (primarily middle and inferior frontal gyri) (4) Temporal lobes (5) Precuneus (6) Insula (7) Hippocampus and parahippocampal gyrus.	Increased maternal empathic abilities; Increased quality of mother-to-infant attachment; Reduced likelihood of maternal hostility toward the infant; Influences maternal motivation and behavior.	Partial volume recovery observed within 3–4 months postpartum (renormalization process); Increased GM volume in: (1) Precuneus (2) Superior temporal gyrus (3) Inferior frontal and medial prefrontal gyrus (4) Anterior cingulate (5) Hippocampus and parahippocampal gyrus (6) Insula (7) Middle occipital gyrus and posterior cerebellum (8) Nucleus accumbens (9) Caudate; 3. Increased GM volume of regions influenced by maternal–infant bonding: (1) Hypothalamus (2) Amygdala (3) Substantia nigra (4) Globus pallidus.
White matter	Enhanced microstructural integrity: (1) Corpus callosum (2) Arcuate fasciculus (3) Inferior fronto-occipital fasciculus (4) Inferior longitudinal fasciculi (5) Cingulum bundle (6) Middle and superior longitudinal fasciculi (7) Corticostriatal tract (8) Corticospinal tract (9) Corticopontine tract; 2. Volume increases in: (1) Frontal lobes (2) Temporal lobes (3) Superior parietal regions (4) Thalamus (5) Caudate nucleus (6) Cerebellum.	Greater tract integrity; Facilitates communication between emotional and visual processing hubs.	Returning to baseline levels

**Table 2:** Impact of Pregnancy on Existing Ophthalmic Disorders.

Ophthalmic Disorders	Disease Activity During Pregnancy	Underlying Mechanisms	Effects in Neuro-Ophthalmic Symptoms
Pituitary adenoma	Enlargement of pre-existing adenomas	1. Lactotroph hyperplasia driven by estrogen and progesterone; 2. Estrogen receptor expression of the pituitary adenoma.	1. Visual disturbances resulting from optic chiasm compression; 2. Headache.
Meningioma	More aggressive behavior	1. Estrogen/progesterone-driven tumor growth; 2. VEGF-mediated vascularization; 3. Predominantly parasellar localization and clear cell or chordoid subtypes.	1. Optic nerve compression; 2. Visual impairment.
Migraine	1. Generally improved; 2. Aura worsened in subset patients.	1. Estrogen fluctuations altering cortical excitability; 2. Cortical spreading depression.	1. Reduced migraine frequency overall; 2. Increased aua frequency in subset of patients.
Diabetic retinopathy	Progression	1. Increased cardiac output and impaired autoregulation causing vascular dilation; 2. Placental hormones (IGF, placental GH) induced angiogenesis; 3. Enhanced inflammation.	1. Progression from non-proliferative to proliferative retinopathy; 2. Increased diabetic macular edema.
Neuromyelitis optica spectrum disorder	Increased disease activity	1. High placental expression of AQP4; 2. Anti-AQP4 antibody-mediated placental inflammation.	1. Exacerbations of optic neuritis and transverse myelitis; 2. Increased miscarriage risk; 3. Increased relapse postpartum.
Multiple sclerosis	Decreased disease activity	1. Immunosuppressive Th2 dominance; 2. Postpartum immunologic rebound.	1. Reduced optic neuritis attacks during pregnancy; 2. Increased postpartum relapse frequency.
Graves’ disease and thyroid orbitopathy	Initial aggravation; subsequent improvement	1. Pregnancy-induced immune modulation; 2. hCG-induced thyroid stimulation; 3. Fluctuating thyroid receptor antibody (TRAb) levels.	Inconsistent association with the severity of ocular symptoms

Vascular endothelial growth factor (VEGF); insulin-like growth factor (IGF); placental growth hormone (GH); Aquaporin-4 (AQP-4); human chorionic gonadotropin (hCG).
